# Differential Expression of the Activator Protein 1 Transcription Factor Regulates Interleukin-1ß Induction of Interleukin 6 in the Developing Enterocyte

**DOI:** 10.1371/journal.pone.0145184

**Published:** 2016-01-22

**Authors:** Catherine M. Cahill, Weishu Zhu, Elias Oziolor, Yao-Jong Yang, Bosco Tam, Susruthi Rajanala, Jack T. Rogers, W. Allan Walker

**Affiliations:** 1 Neurochemistry Laboratory, Department of Psychiatry, Massachusetts General Hospital, and Harvard Medical School, Charlestown, Massachusetts, United States of America; 2 Mucosal Immunology and Biology Research Center, Department of Pediatrics, Massachusetts General Hospital for Children, and Harvard Medical School, Charlestown, Massachusetts, United States of America; 3 Department. of Environmental Science, Baylor University One Bear Place #97266, Waco, Texas, United States of America; 4 Departments of Pediatrics and Internal Medicine, National Cheng Kung University, Tainan, Taiwan; Dana-Farber/Harvard Cancer Institute, UNITED STATES

## Abstract

The innate immune response is characterized by activation of transcription factors, nuclear factor kappa B and activator protein-1 and their downstream targets, the pro-inflammatory cytokines including interleukin 1β and interleukin 6. Normal development of this response in the intestine is critical to survival of the human neonate and delays can cause the onset of devastating inflammatory diseases such as necrotizing enterocolitis. Previous studies have addressed the role of nuclear factor kappa B in the development of the innate immune response in the enterocyte, however despite its central role in the control of multiple pro-inflammatory cytokine genes, little is known on the role of Activator Protein 1 in this response in the enterocyte. Here we show that the canonical Activator Protein 1 members, cJun and cFos and their upstream kinases JNK and p38 play an essential role in the regulation of interleukin 6 in the immature enterocyte. Our data supports a model whereby the cFos/cJun heterodimer and the more potent cJun homodimer downstream of JNK are replaced by less efficient JunD containing dimers, contributing to the decreased responsiveness to interleukin 1β and decreased interleukin 6 secretion observed in the mature enterocyte. The tissue specific expression of JunB in colonocytes and colon derived tissues together with its ability to repress Interleukin-1β induction of an Interleukin-6 gene reporter in the NCM-460 colonocyte suggests that induction of JunB containing dimers may offer an attractive therapeutic strategy for the control of IL-6 secretion during inflammatory episodes in this area of the intestine

## Introduction

Intestinal epithelial cells (IEC) play an important role in mucosal immunity, a developmental program which continues throughout gestation into the *post partum* period. Fetal enterocytes express receptors and signaling molecules that are involved in the innate immune response including toll like receptors, including TLR4 [1] [2,3] and the interleukin 1 receptor (IL-1R) which can induce potent local acute inflammatory responses resulting in elevated production of the pro-inflammatory cytokines interleukin 6 (IL-6) and interleukin 8 (IL-8).

Abnormal maturation of the intestinal mucosal immune response in infants can lead to illness such as necrotizing enterocolitis (NEC) an inflammatory disease which afflicts the neonatal ileum and proximal colon and is associated with prematurity [4] [5] [6]. This hyper-inflammatory condition leading to intestinal necrosis and perforation affects up to 13% of very low birth weight infants in the U.S. with a mortality as high as 50% of those requiring surgery [5]. A hallmark of NEC is a developmental immaturity of specific innate immune response genes including components of the TLR4 and IL-1R pathways resulting in prolonged NFκB activation and secretion of pro-inflammatory cytokines such as IL-6 and IL-8 [7,8]. Transcription of many pro-inflammatory cytokine genes are controlled by nuclear factor kappa B (NFĸB) including IL-6 and IL-8 and both IL-6 and IL-8 are elevated in NEC with elevated IL-6 being an indicator of poor prognosis in afflicted infants [9]. Reports have shown developmental differences in the responses to IL-6 in rodents where IL-6 transgenic mice exposed to hyperoxia have increased mortality due to DNA injury and cell death in the neonatal period [10]. IL-6 is associated with gut barrier dysfunction [11,12]. Antibody neutralization of IL-6 has been shown to improve gut function and lessen severity of disease. Understanding IL-6 gene regulation in this sensitive neonatal period will help to control its secretion during inflammatory episodes.

Interleukin-6 gene transcription is regulated mainly at the level of transcription with important roles for both NFĸB and activator protein 1 (AP-1) [13]. Both NFĸB and AP-1 are activated downstream of IL-1β and cross-talk between these have been reported to contribute to transcription of the IL-6 gene in the Caco-2 enterocyte where we identified AP-1 as one of the necessary factors regulating its’ transcription [14].

Activator-Protein 1 is a leucine zipper transcription factor composed of homo or hetero dimers of members of the Jun, Fos and ATF proteins [15]. There are three distinct Jun proteins, i.e., c-Jun, JunB, and JunD, and four Fos members, i.e., c-Fos, FosB, Fra1, and Fra2. AP-1 activates transcription by binding to the canonical AP-1 response element, a DNA recognition element (5'-TGAG/CTCA-3') which also mediates transcriptional induction in response to the tumor promoter, TPA[16]. AP-1 is controlled at multiple levels including gene transcription, mRNA turnover, protein stability, interactions with other leucine zipper factors, posttranslational modifications as well as by signaling from upstream Map kinases[16]. Phosphorylation of cJun on ser-63/73 by the Map kinase, Stress activated protein kinase, SAPK, JNK mediates its’ transcriptional activation [17,18]. Transcriptional control of Fos is mediated by ERK and p38 [19]. Developmental expression patterns of some AP-1 family members, including cJun and JunB in the rodent is suggestive of a role in cellular differentiation [20]. In the Caco-2 enterocyte, induction of AP-1 DNA binding is likely involved in the switch from proliferation to differentiation. Several members of the AP-1 family have been shown to regulate the IL-6 gene. JunB has been shown to control the IL-6 gene in skin fibroblasts and in T cells [21,22]. JunD, has been shown to control IL-6 gene transcription in response to TGFβ in lung fibroblasts [23] and cJun has been shown to co-operate with NFĸB and other transcription factors in IL-6 induction in response to TNFα in Hela cells [24].

We have previously used the established human fetal small intestinal xenograft model of human intestinal development to characterize the ontogeny of the innate immune system throughout a 10 month gestation period [25]. Fetal human gut is transplanted subcutaneously into immune-compromised mice (SCID) and has the capability of regenerating and surviving for extended periods of time. Following transplantation, this tissue undergoes initial degeneration followed by angiogenesis and re-epithelialization of the luminal surface and does this as if *in utero*. It regenerates with an appropriate spatiotemporal organization of crypt-villus axis and with similar functional maturation including sucrase and lactase production as observed *in utero [26]*. Crypts and villi appear by 8 and 10 weeks respectively [27] and these developed xenografts are classified as having differentiated mucosa corresponding to the equivalent age fetal intestine. At 24 weeks of gestation, hydrolase activity, a useful tool to delineate the functional ontology of the small intestine and is associated with maturation, is comparable to that of newborn levels. Using this xenograft model together with our non-transformed immature H4 enterocyte we have defined several genes responsible for the ontogeny of the mucosal immune system including IĸBα, which by sequestering NFκB in the cytoplasm leads to decreased NFĸB activation thereby lowering the expression of pro-inflammatory mediators such as IL-8 during the maturation period. We have also shown that the expression of TLR2, TLR4, MyD88, TRAF-6, NFkappaB1 and IL-8 mRNA are increased while negative regulators of inflammation, SIGIRR, IRAK-M, A-20 and TOLLIP mRNA were decreased in fetal vs. mature human enterocytes [8]. We showed that cortisone, an intestinal maturation factor, corrected the mRNA differences resulting in decreased of pro-inflammatory cytokines [26].

To our knowledge there are no reports of AP-1 family expression in the neonatal *human* intestine. There are few reports of IL-6 secretion by the immature enterocyte and none of these address IL-6 gene regulation directly or the role of AP-1[28–30]. The aim of this study was to investigate the role of Stress activated protein kinase/AP-1 pathway in the control of IL-6 secretion in the developing enterocyte. Comparing our immature and mature non transformed human enterocyte models, H4 and NCM-460 enterocytes and immature and mature intestinal xenografts we demonstrate the importance of this pathway, in particular JNK/cJun in the control of IL-6 production in response to IL-1β the immature enterocyte.

## Materials and Methods

### Cell lines

The H4 cell is a non transformed ileal crypt enterocyte derived from a 22 week old human fetal ileum isolated in this laboratory and was cultured in Dulbecco’ modified Eagle’s medium with 10% fetal bovine serum (0.05 units endotoxin), insulin, EGF and antibiotics (100 mg/liter) streptomycin and penicillin as described previously [31]. The NCM460 cell line (Incell Corporation, LLC) is a non transformed colon enterocyte derived from mature colon and is a mix of both mesenchymal and epithelial cells and are a mixed population of cells representing cells in various stages of differentiation. NCM-460 cells were grown in conditioned medium (25–50% V/V) (M3:DC Defined medium) ([32].

### Xenograft organ culture model

#### Fetal tissue

Human fetal intestinal samples were obtained from elective prostaglandin/saline- induced therapeutic abortion of 12–16 week fetuses, after informed written consent. (Protocol 1999-P-003833 [Walker]) and according to the regulations of the Committee for the Protection of Human Subjects from Research Risks at the Brigham and Women’s Hospital and the Human Investigation Committee at Massachusetts General Hospital. The study was conducted according to the National Institutes of Health (NIH, Bethesda, MD) guidelines. All experiments involving and utilizing human tissues were approved by the Institutional Review Board for the Massachusetts General Hospital, (Protocol 1999-P-003833, Walker).

Sterile fetal small intestinal, cecal and colon segments (2cm) were implanted subcutaneously into SCID mice as described previously [27,33]. Because complete structural and functional maturation of the small intestine occurs after 38 weeks of gestation we harvested xenografts prior to and beyond this time, to represent immature, mature and post-natal intestine [25]. Xenografts were harvested at 16, 24, 28 and 44 weeks post-transplantation and were cut into 5X5mm explants for deposit onto Falcon organ culture dishes and maintained in culture media at 37°C in 95%O2/5%Co2. Xenografts were grown in CMRL1066 supplemented with glucose (5g/L), methionine (1ug/L), tricine buffer (20mM, pH 7.4), hydrocortisone hemisuccinate (0.5ug/L), β, retinyl acetate (1mg/L), glutamine (3mM/L), 5% FBS (HyClone), penicillin G (100 units/L) and gentamicin (50mg/L). Explants were cultured from each xenograft and allowed to equilibrate for at least 6h before treatment with IL-1β.Three explants derived from 3 individual age matched xenografts were either untreated or treated overnight with IL-1β (1ng/ml). Media and tissue were then collected and stored at -80. Sucrase activity was determined to assess morphological integrity of the mucosa and structural viability of the organ culture. In addition several explants from organ cultures were subject to histological sections to assess structural integrity of the mucosa. The capacity of IL-1β to induce IL-6 was quantitated by ELISA assay and expressed as ng/mg of total tissue protein.

### Pharmacological inhibitors and Interleukin 6 assay

The JNK inhibitor, SP600125 and the p38 inhibitor SB203580 (Calbiochem) and the IKK inhibitor (Wedelolactone) were used at or below their IC50 values. Cell and organ cultures were incubated with inhibitors 15 minutes prior to IL-1β treatment (0.1ng/ml). IL-6 was assayed by ELISA (R and D Systems, MN).

### Nuclear Extract preparation and Activator Protein 1 binding to DNA

Nuclear extracts were prepared using the NE-PER nuclear and cytoplasmic extraction reagents as described by the manufacturer (Pierce Biotechnology, Thermo Scientific). AP-1 subunit binding to the canonical AP-1 site (5’-TGAGTCA-3”) from nuclear extracts derived from H4 cells (2.5ug) and NCM460 cells (5ug) were analyzed using the TransAM ELISA kit using specific antibodies to Fos family members (cFos, Fra 1 FosB) and Jun Family members (cJun, JunB and JunD). Briefly, nuclear extract (containing activated transcription factor) was added to oligonucleotide-coated wells. After 20 min of incubation at room temperature, plates are washed, and incubated with diluted specific antibodies to AP-1 members for 1h, washed and incubated further with HRP-conjugated secondary antibody and substrate.

### Western Blot Analysis

Western analysis was carried out as described previously [14]. Harvested cells and tissues were homogenized in ice cold lysis buffer (Pierce) supplemented with protease inhibitors. All lysates were assayed for total protein using a BCA® Protein assay kit with BSA as standard. Protein was loaded on a 10–20% SDS gel. Antibodies used were as follows, Santa-Cruz; Rabbit anti-cFos(4):sc-52, rabbit anti-JunB (N-17):sc-46, rabbit antiJunD (329):sc-74; anti-rabbit Fra1 (R20):sc-605; anti rabbit FosB (102):sc-48, rabbit total JNK (sc-474). Cell Signaling; Rabbit monoclonal phospho cJun (ser 63), rabbit phospho p38 (Thr180/Tyr182), rabbit anti- p38. Invitrogen; rabbit monoclonal phospho JNK 1/2 (pT183/pY185), monoclonal anti-beta actin (clone AC-15); Amersham ECL sheep Anti-Mouse IgG, HRP; donkey ECL anti rabbit IgG HRP. Blots were developed with chemiluminescence (Pierce) and visualized with a PhosphorImager. Bands were quantified using Quantity One software (Bio-Rad).

### Transfection and reporter assays

The *pIL-6*-luc651 plasmid, containing a 651-bp fragment of the human *IL-6* gene promoter located directly upstream of the transcription start site, and the 2 mutated plasmids, *pIL-6*-651mAP-1, and *pIL-6*- 651mNFkB were a generous gift from Dr. Oliver Eickelberg, University Hospital, Basel, Switzerland[23,34]. Transcription factor binding site mutations were AP-1-283 to 276, 5’-TGAGTCAC-3’ was changed to ‘5-TGCAGCAC- 3’; and the NFkB consensus sequence from -72 to -63, 5’-GGGATTTTCC-3’, was changed to 5’-CTCATTTTCC-3’. These mutations have previously been shown to abrogate transcription factor binding. The AP-1 reporter plasmid was a gift from Michael Greenberg, Children’s Hospital Boston. It is a 5X repeat of the canonical TPA response element (3’-TGAGTCAC-5’). The NFkB reporter plasmid is from the IgĸB NFkB site (LMMP, University of Ghent, Belgium). Expression plasmids for cJun and Tam67 and JunB were obtained from Dr. Mark Piechaczyk and Dr. Johan Garaude, Institut de Genetique Moleculaire du Montpellier, France) JunD and JunD MT were obtained from Dr. Joel Habener (MGH). H4 and NCM460 cells were plated to 50% confluence in transfection media (growth media minus antibiotics) in 24-well plates. After overnight attachment, cells were transfected with firefly luciferase reporter plasmids (0.25ug) in the presence or absence of expression plasmids (0.125 ug/well) together with a control renilla luciferase reporter plasmid (0.025ug) for estimation of transfection efficiency. Eighteen hours after transfection, cells were serum starved for 6h and were either left untreated (C) or treated overnight with IL-1β (0.5 ng/ml). All plates were harvested 18 h after treatments and assayed for luciferase activity (Dual-Luciferase reporter assay, Promega). Results are expressed as normalized values, firefly luciferase/ renilla luciferase.

### Statistical Analysis

Results are presented as means+/-SE. Experimental points were performed in triplicate with three independent experiments (N = 3). Statistical comparisons between control and experimental groups were by two way ANOVA in conjunction with Sidan’s test for multiple comparisons. Students t- test was routinely used for pairwise comparisons. Differences with a P value of ≤0.05 were considered significant.

## Results

### Basal and IL-1β induction of IL-6 is higher in immature enterocytes and intestinal epithelium compared to their mature counterparts

**Fig 1:**
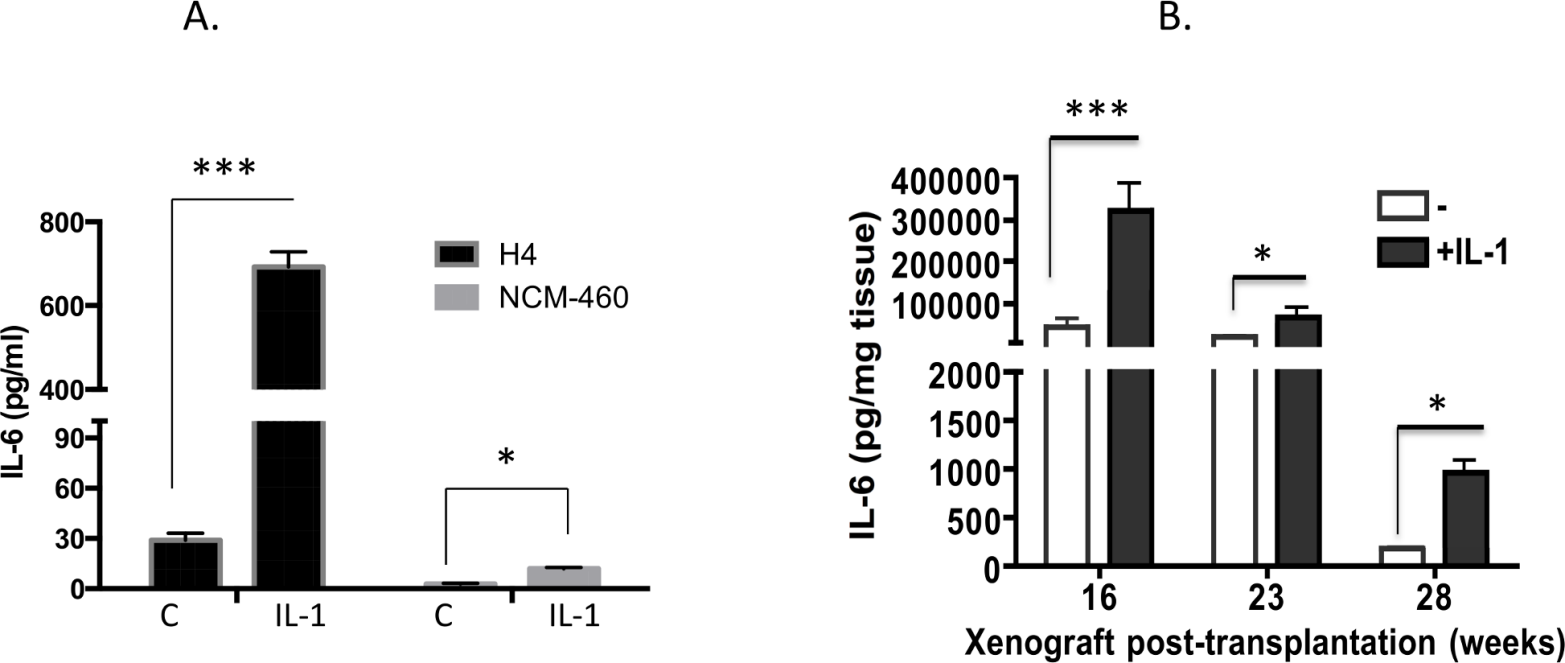
Interleukin 1 induction of IL-6 in immature and mature enterocytes and in developing ileal xenografts. **(A) IL-1**b **induction of IL-6 in immature H4 and in mature NCM460 enterocytes.** Cell lines were plated in 24 well plates overnight, incubated in low serum media for 3h and treated with IL-1β (0.5ng/ml). Tissue culture media was harvested after 6 h and assayed for IL-6 by ELISA Mean +/-S.E. (n = 3) from 3 experiments are presented ***p<0.001, *p<0.05 **(B). IL-1**β **induction of IL-6 in human ileal xenografts grown *ex vivo* in organ culture.** Human ileal explants derived from xenografts transplanted subcutaneously into the SCID mouse and harvested 16, 23 and 28 weeks post-transplantation were equilibrated for up to 6h in organ culture media and were either untreated or treated with IL-1b(0.5ng/ml). Tissue culture media was harvested for IL-6 measurement after 24h. Mean +/-S.E. (n = 3) from 2 experiments are presented ***p<0.001, *p<0.05.

In Fig 1 we compare IL-6 secreted in immature H4 ileal enterocytes and mature NCM460 colon derived enterocytes. Both of these cell lines are non transformed human enterocytes which are better cell line models of normal human enterocytes than cancer cell models. Basal IL-6 production is 20-fold higher in immature H4 cells compared to the mature NCM-460 cells and IL-1β induces a 30-fold increase in IL-6 production compared to only 5-fold induction in mature NCM460 cells (Fig 1A). In Fig 1B we investigate IL-6 production by immature (16 week) and mature (23, 28 week) ileal xenografts in organ culture. We demonstrate that there is 25-fold higher basal IL-6 production in the immature ileal cultures compared to the mature. Baseline IL-6 drops precipitously by 28 weeks. There is an 8-fold induction of IL-6 by IL-1β in the immature xenograft at 16 weeks which drops to between 4–5 fold as the xenografts mature. Taken together, this shows that immature intestinal epithelium secretes IL-6 constitutively and responds with a stronger IL-1β response than its mature counterpart.

### Interleukin 1β induces IL-6 secretion in immature H4 enterocytes *via* the Stress Activated Protein Kinases (SAPK) JNK and p38

**Fig 2:**
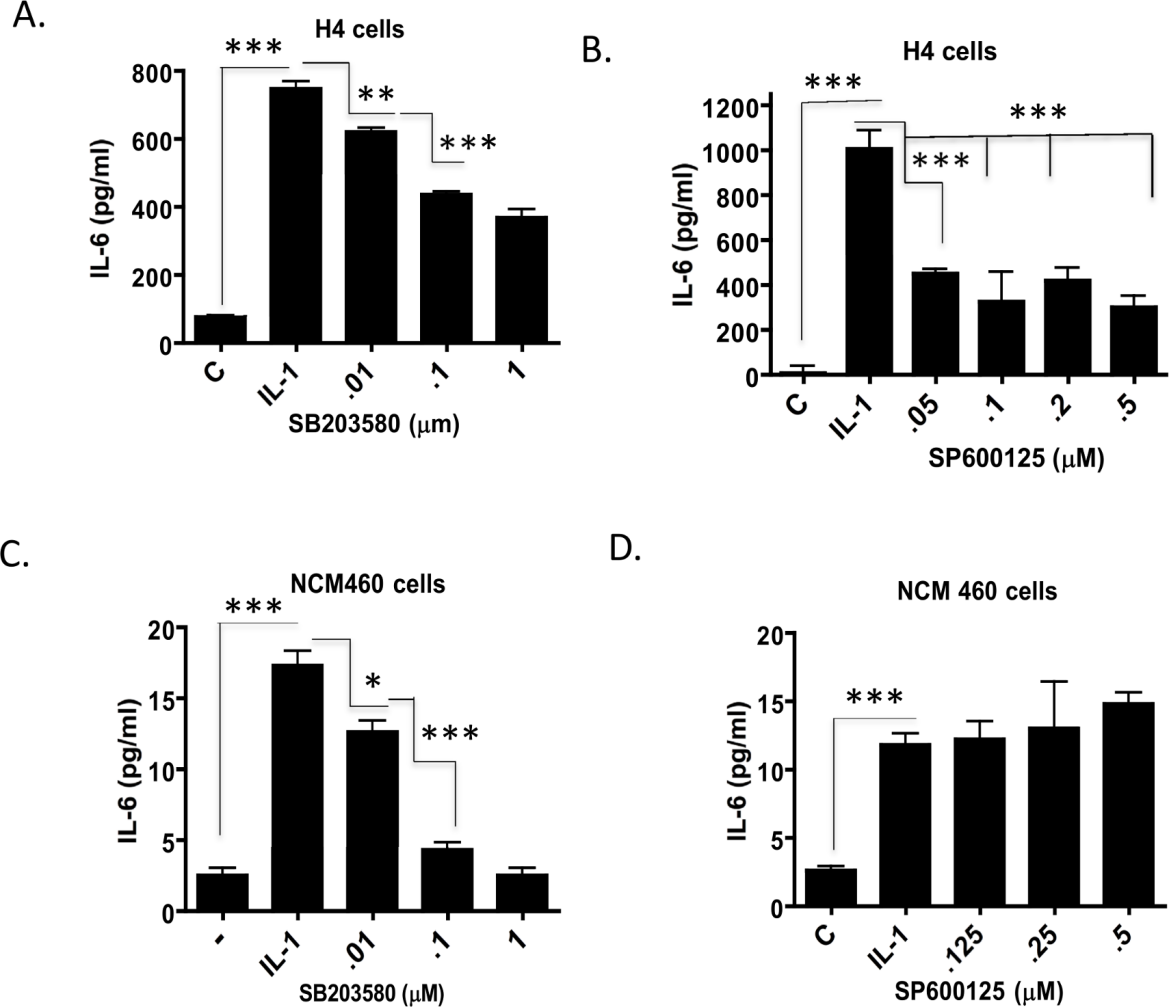
Interleukin 1β induction of IL-6 is mediated by the Stress Activated Protein Kinases, JNK and p38 in immature H4 enterocytes. Immature H4 cells (A, B) and mature NCM-460 cells (C, D) were plated in 24 well plates overnight, incubated in low serum media for 3h and treated with IL-1β (0.5ng/ml) in the absence or presence of the JNK inhibitor SP600125 and the p38 inhibitor SB203580 (triplicate treatments) as indicated. Tissue culture media was harvested after 6 h and assayed for IL-6 by ELISA. Mean +/-S.E (n = 3) from 2 experiments are presented. Control un induced compared to IL-1 and IL-1 induced compared to IL-1 in the presence of inhibitors, ***p<0.001, **p<0.01 *p<0.05) analyzed by Student T-test.

Our previous studies have demonstrated that the ERK Map kinase is an important signaling cascade downstream of IL-1β in the induction of IL-6 in the immature enterocyte [29]. This prompted an investigation on the role of the Stress Activated Protein Kinases, a parallel Map kinase pathway activated by stress and the pro-inflammatory cytokine IL-1β. To investigate its’ role in the induction of IL-6 by IL-1β in the non transformed immature and mature enterocytes we pretreated immature H4 and mature NCM460 cells with the SAPK inhibitors SP-600125 (JNK inhibitor) and separately with SB203580 (the p38 SAPK inhibitor) prior to IL-1β treatment. Pharmacological inhibition of both JNK and p38 SAPK significantly impairs IL-1β induction of IL-6 in immature H4 cells (Fig 2A and 2B) with the JNK inhibitor having a greater inhibitory effect. Contrasting this, in the mature NCM-460 enterocytes, only the p38 inhibitor, SB203580, inhibits IL-1β induction of IL-6 with no effect of the JNK inhibitor, SP600125 (Fig 2C and 2D). At a dose level of 1μM of SB203580, IL-1 induction of IL-6 is completely abrogated by SB203580 in NCM460 cells while it is only 50% impaired in H4 cells while the JNK inhibitor SP600125 as low as 0.1uM impairs IL-6 secretion by 70% with no affect in NCM460 cells. Taken together, this suggests that the JNK pathway is more important in immature H4 enterocytes and p38 is more important in the mature enterocytes in mediating IL-1**b** induction of IL-6.

### Elevated basal JNK and p38 phosphorylation is increased by IL-1β in the immature enterocyte

**Fig 3:**
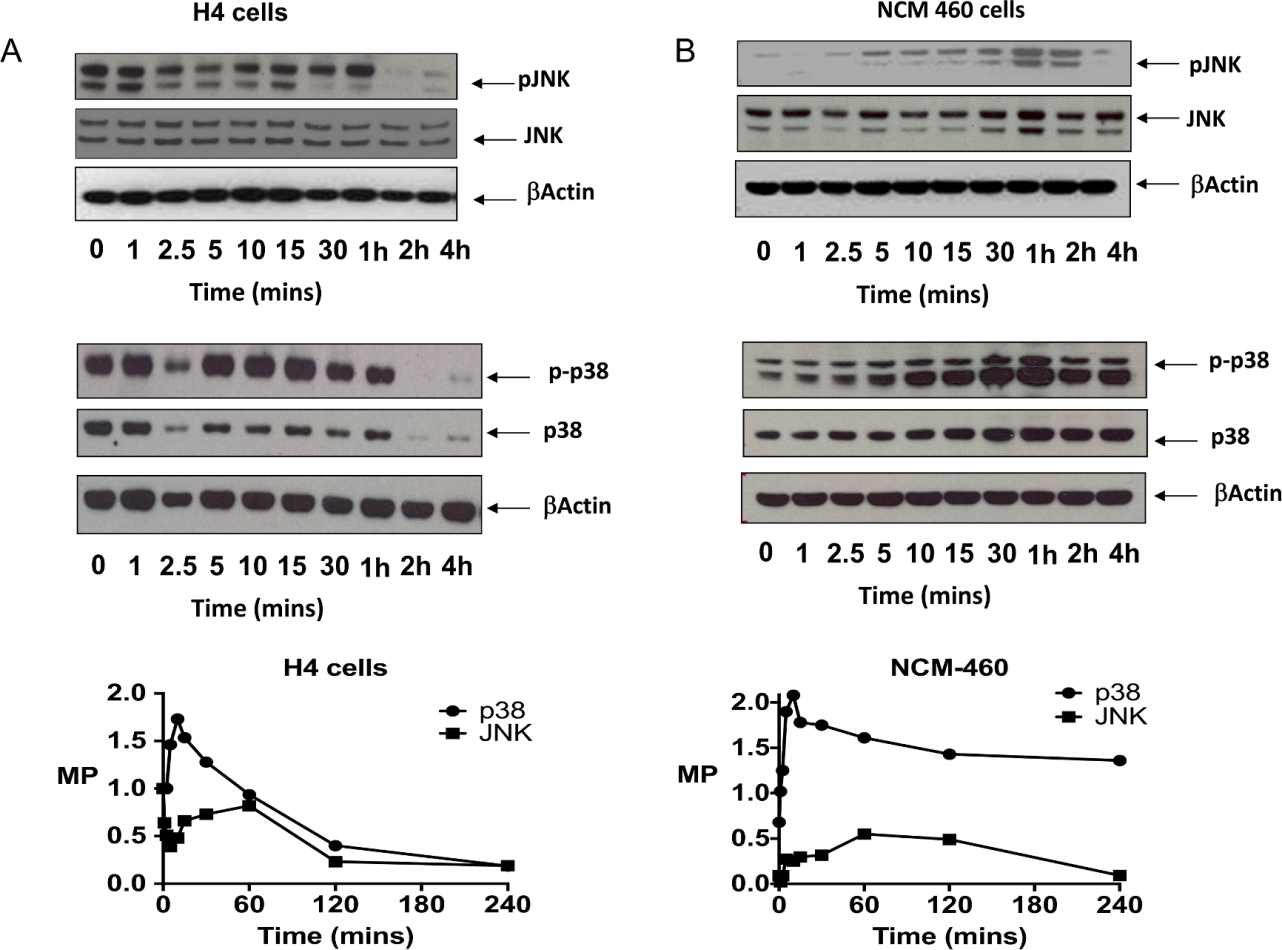
Interleukin 1β induction of JNK and p38 phosphorylation in immature H4 and mature NCM-460 enterocytes. Western analysis of JNK and p38 phosphorylation in immature H4 enterocytes (A) and mature NCM-460 enterocytes (B) in response to IL-1β. Cells were grown in 10 cm dishes to 90% confluence, incubated in low serum media for 3h followed by exposure to IL-1β (0.5 ng/ml). Cells were harvested at the indicated time points and lysates (15ug) run on SDS PAGE (4–12%) and blotted with the indicated antibodies. Bands were quantitated using quantity 1 software. Molar phosphorylation, calculated as the ratio of the density of the phosphorylated bands to the unphosphorylated bands are presented (lower panels). A representative experiment of 3 separate experiments is presented.

We next compared phosphorylation status of JNK and p38 in the H4 and NCM460 enterocytes. Phosphorylation of JNK on Thr183/Y185 and p38 on Thr180/Y182 reflect their levels of cellular activation [35]. In Fig 3A, a time course experiment in H4 cells reveals high constitutive phosphorylation of JNK which initially decreased upon IL-1β exposure and later increased from the 10- 60min. Thereafter the phosphorylation of JNK declined to undetectable levels. High basal phosphorylation of p38 was also detected in H4 cells and was further increased at the earlier 15 min time point by IL-1β stimulation. Phosphorylated JNK and p38 were almost undetectable at 2h in H4 cells. Contrasting this in mature NCM460 enterocytes, basal JNK phosphorylation was very low and increased 5-*fold* with exposure to IL-1β to maximum detectable levels at 1h declining thereafter to undetectable levels. In NCM460 cells there is also low basal phosphorylation of p38 which was increased by IL-1β and sustained for up to 4h (Fig 3B, lower panel).

### Binding of Activator Protein 1 components to the canonical TPA response element in response to IL-1β in immature H4 and mature NCM-460 enterocytes

The canonical family members of the AP-1 transcription factor family, cJun and cfos are major downstream targets of the SAPKs. We employed the TRANS-AM ELISA to analyze the AP-1 family components which bind to the canonical AP-1 response element in response to IL-1β in nuclear extracts derived from H4 and NCM460 cells. The TRANS-AM ELISA, uses TRE DNA coated wells and antibodies to detect specific members of the AP-1 family and is a more sensitive DNA binding assay than the conventional electrophoretic mobility shift assay.

**Fig 4:**
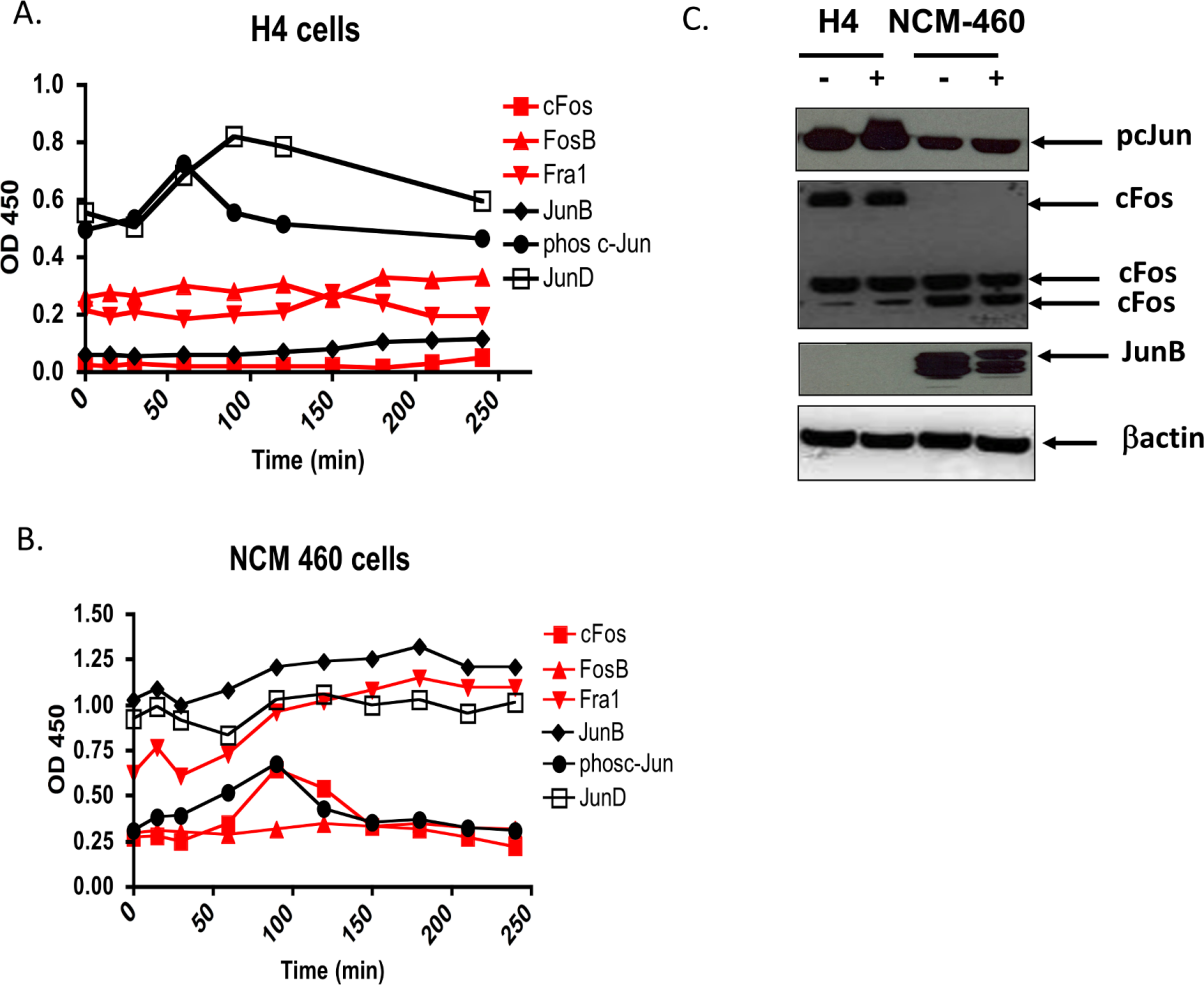
Binding of Activator Protein 1 family members to the TPA response element in nuclear extracts derived from enterocytes exposed to IL-1β Nuclear extracts were prepared from H4 cell lysates (A) and NCM 460 cell lysates (B) and were assayed for binding to the TPA response element using the TRANS-AM ELISA. (black: Jun Family members; red: Fos family members,). Cells were grown in 10 cm dishes to 90% confluence, incubated in low serum media for 3h followed by exposure to IL-1β (0.5 ng/ml) for the indicated times. Cells were harvested at T0, and every 30 minutes thereafter for up to 4h. Whole cell lysates derived from H4 and NCM460 cells (C) were analyzed by western blotting using specific antibodies for phosphorylated cJun, cFos and JunB. Experiments were performed three times with consistent results. A representative experiment is presented.

Confluent H4 and NCM-460 cells were exposed to IL-1 and cells were harvested at time 0 and every 30 mins thereafter for up to 4 hours. Nuclear extracts from H4 cells demonstrate a pre-dominance of Jun family binding. Constitutive cJun and JunD binding and a notable absence of JunB and cFos binding is observed (Fig 4A) IL-1b induces a transient increase in the binding of cJun at 1h, correlating with maximal JNK phosphorylation (Fig 3A). The binding of JunD increases in parallel with cJun but increases further past the 60 min time point and is maximal between 90–120 mins suggesting that the activating dimer in H4 cells is likely a potent cJun homodimer. There was low, but detectable Fra1 and FosB binding which increased slightly in response to IL-1.

In the mature NCM 460 colonocyte the binding of the canonical cJun, cFos was low initially with phospho cJun increasing gradually to the 90min time point where cFos binding was also highest. In contrast to the absence of binding of JunB in H4 nuclear extracts, there was elevated constitutive binding of JunB in NCM 460 cells and the pattern of JunB and JunD as well as Fra1 followed a similar binding pattern in response to IL-1β.

To investigate if the absence of detectable JunB and cFos binding in H4 cells was due to their absence in the lysates we performed western analysis on whole cell lysates derived from H4 cells and compared them to lysates from the NCM-460 cell. We detected strong expression of JunB in the colon derived NCM-460 cells while there was no detectable expression of JunB in the H4 ileal cells (Fig 4C). The upward mobility shift of the highly expressed JunB in NCM460 cells suggests post translational modifications in response to IL-1b. H4 cells expressed high levels of cJun and cFos although much of the cFos was expressed as a high molecular weight band suggesting it is expressed as a precursor form or has post translational modifications that may prevent it from binding DNA.

### Interleukin 1b induces IL-6 secretion *via* the SAPKs JNK and p38 in immature human ileal xenografts

**Fig 5:**
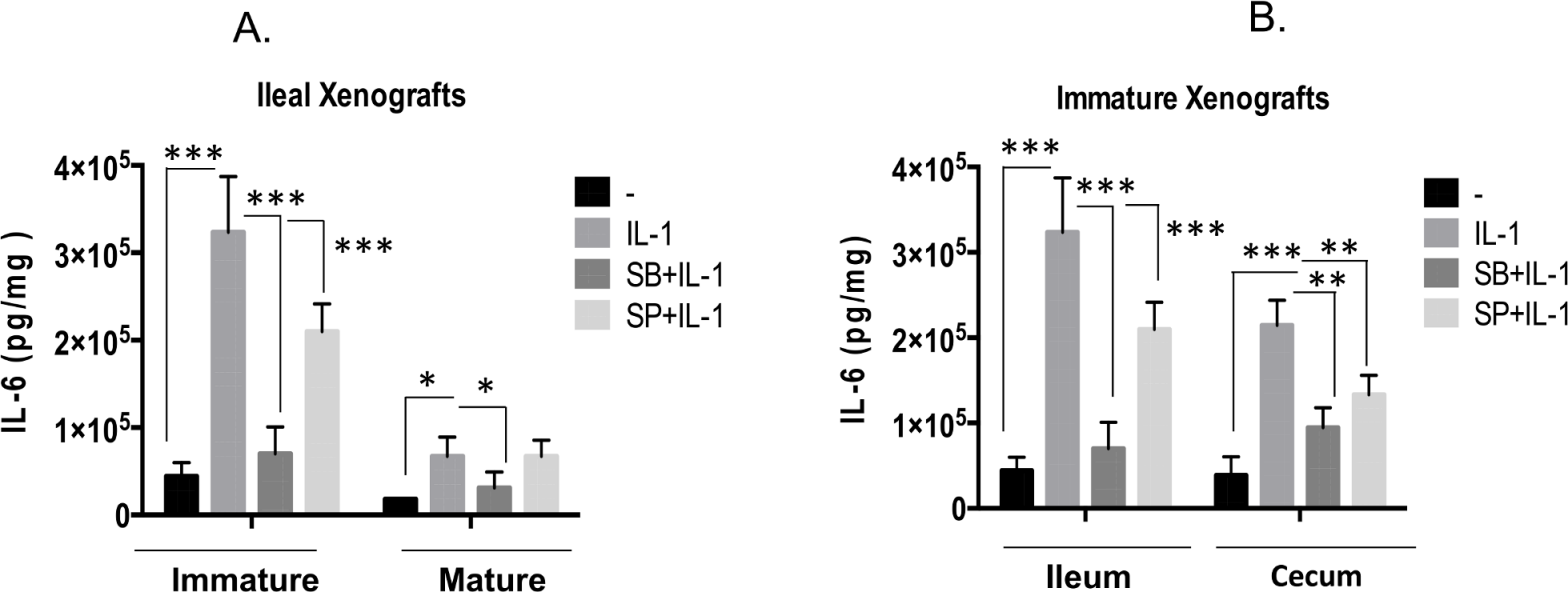
Interleukin 1β induction of IL-6 secretion by immature intestinal xenografts is mediated by the Stress Activated Protein Kinases JNK and p38. In A, Xenografts from immature and mature ileum (23, 28 post transplantation) and (B) immature ileum and cecum 16 weeks post transplantation) were cultured as explants in organ culture media. Following equilibration for 3h, explants (in triplicate) were either untreated or exposed to IL-1b (0.5ng/ml) in the presence or absence of the JNK inhibitor SP600125 (0.2υM) or the p38 inhibitor SB203508 (1uM). Tissue culture media was harvested after 24h and assayed for IL-6 by ELISA. Data, derived from 3 separate experiments is expressed as Means +/-SE (n = 3) ***p<0.001, **p<0.01 *p<0.05.

Next we investigated the role of the SAPK/AP-1 pathway in our *ex vivo* model of human intestinal development employing human fetal intestinal explants from xenografted tissue from the SCID mouse. Xenografts were harvested, cut into 2-3mm explants and cultured overnight as organ cultures [27]. Using this model we exposed immature ileal and cecal explants (16–23 week post transplantation) and mature ileal explants (28 weeks post transplantation) to IL-1b in the presence or absence of the pharmacological inhibitors of the SAPKs, SB203508 and SP600125 (Fig 5A and 5B). Similar to our data in Fig 1B there was an 8-fold induction of IL-6 by IL-1b in the immature ileal explants compared to a 3-fold induction in the mature. Pharmacological inhibition of p38 with SB203508 almost completely abrogated the response to IL-1β in both immature and mature ileal cultures. Inhibition of JNK significantly inhibited the response in the immature and had no effect in the mature cultures. This is consistent with the *in vitro* cell line data where SP600125 had no effect on IL-1β induction of IL-6 in the mature NCM-460 cell line (Fig 2D). Explants derived from the immature cecum, an area of the intestine also affected by NEC associated inflammation demonstrated a 6-fold induction of IL-6 in response to IL-1. Consistent with the data from the immature H4 cell line and ileal cultures, both of the SAPK inhibitors had a significant inhibitory effect on IL-6 production by cecal explants. Taken together, both JNK and p38 are both necessary for IL-1β induction of IL-6 in the immature intestine and the requirement for JNK is lost as the ileum matures.

### Elevated expression of cFos and cJun in immature ileal xenografts with later expression of JunD

**Fig 6:**
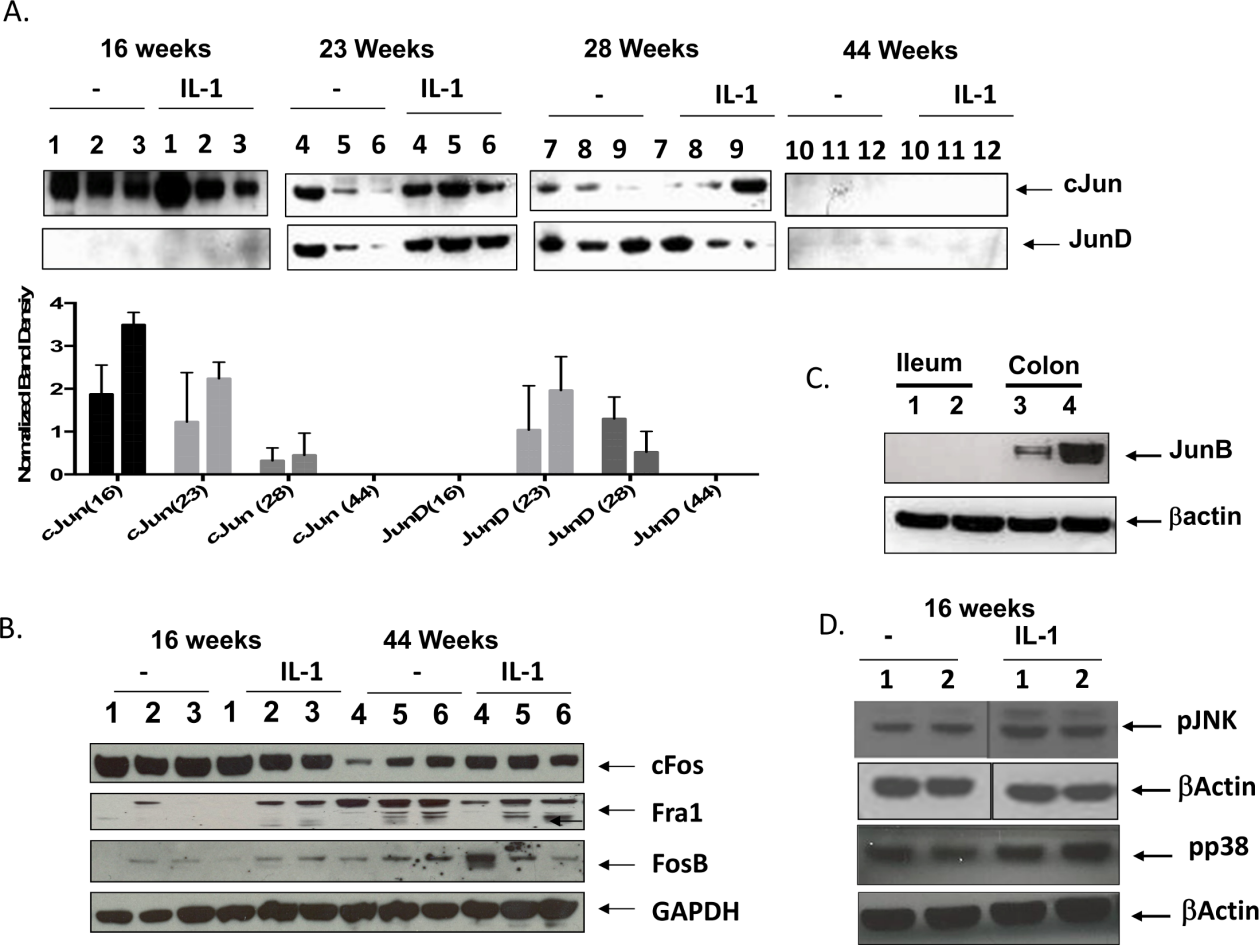
The canonical AP-1 family members, cJun and cFos are highly expressed in the immature ileal xenografts and decrease during maturation. Whole tissue lysates (15ug) from immature xenografts (16 weeks) compared to more mature ileal xenografts (23, 28, 44 weeks) 3 individual mice per developmental stage, 12 mice total. Organ cultures from xenografts were equilibrated in organ culture media for 3h and incubated for 18h in the presence or absence of IL-1b. Lysates were western blotted for the presence of cJun (A) and cFos (B) as well as other members, including JunD, Fra1 and FosB and loading control GAPDH. In A (lower panel) densitometry analysis of the developed bands normalized to βActin is presented. Results are expressed as Means+/-SE (n = 3). In C lysates derived from mature colon and ileum were compared for the presence of JunB and in D immature ileal lysates were blotted for the presence of phosphorylated JNK and p38 (D) with loading control, βActin.

In Fig 6 we examine the AP-1 family member composition in whole tissue lysates derived from immature and mature ileal xenografts grown overnight in organ culture. Expression of cJun and cFos was high in immature xenograft lysates and IL-1β increased expression of cJun but not cFos (compare xenografts from 3 separate animals, (# 1–3, Fig 6A and 6B). No JunD was expressed in immature (16 week) xenografts but was expressed in 23/28 week old xenografts with increased expression in response to IL-1β at 23 weeks. Similar to H4 ileal cells, there was a notable absence of JunB expression in all of the ileal xenografts. To confirm the absence of JunB expression in the ileal explants we compared lysates from 2 colon xenografts to 2 ileal xenografts for the presence of JunB. In Fig 6C, lysates derived from similar age (mature) ileal and colon xenografts were blotted with an antibody against JunB and show Jun B expression only in colonic xenografts. This suggests that JunB may only be expressed in the colon. Cell lysates derived from T84 and Caco-2 colon enterocytes also showed elevated expression of JunB (not shown). There was a notable absence of the full length 39kDa cJun band and JunD in the postnatal xenografts (44 weeks) and a significant decrease in cFos expression suggesting that the canonical members of the AP-1 family, cFos and cJun decrease during ileal maturation (Fig 6B). There was a significant increase in other fos family members, Fra1 and FosB in postnatal ileal xenografts. In Fig 6D and similar to our cell line data we demonstrate that immature ileal explants have high basal JNK and p38 phosphorylation that are induced when cultures are exposed to IL-1b. Taken together with the data in Fig 1 the immature xenografts which secrete high amounts of IL-6 in response to IL-1b express high levels of phosphorylated SAPK together with cJun and cFos. Mature xenografts secrete less IL-6 and express lower amounts of cFos and cJun but increased expression of JunD.

### Interleukin 1β induction of the IL-6 gene in the immature enterocyte is dependent on both NFĸB and AP-1

**Fig 7:**
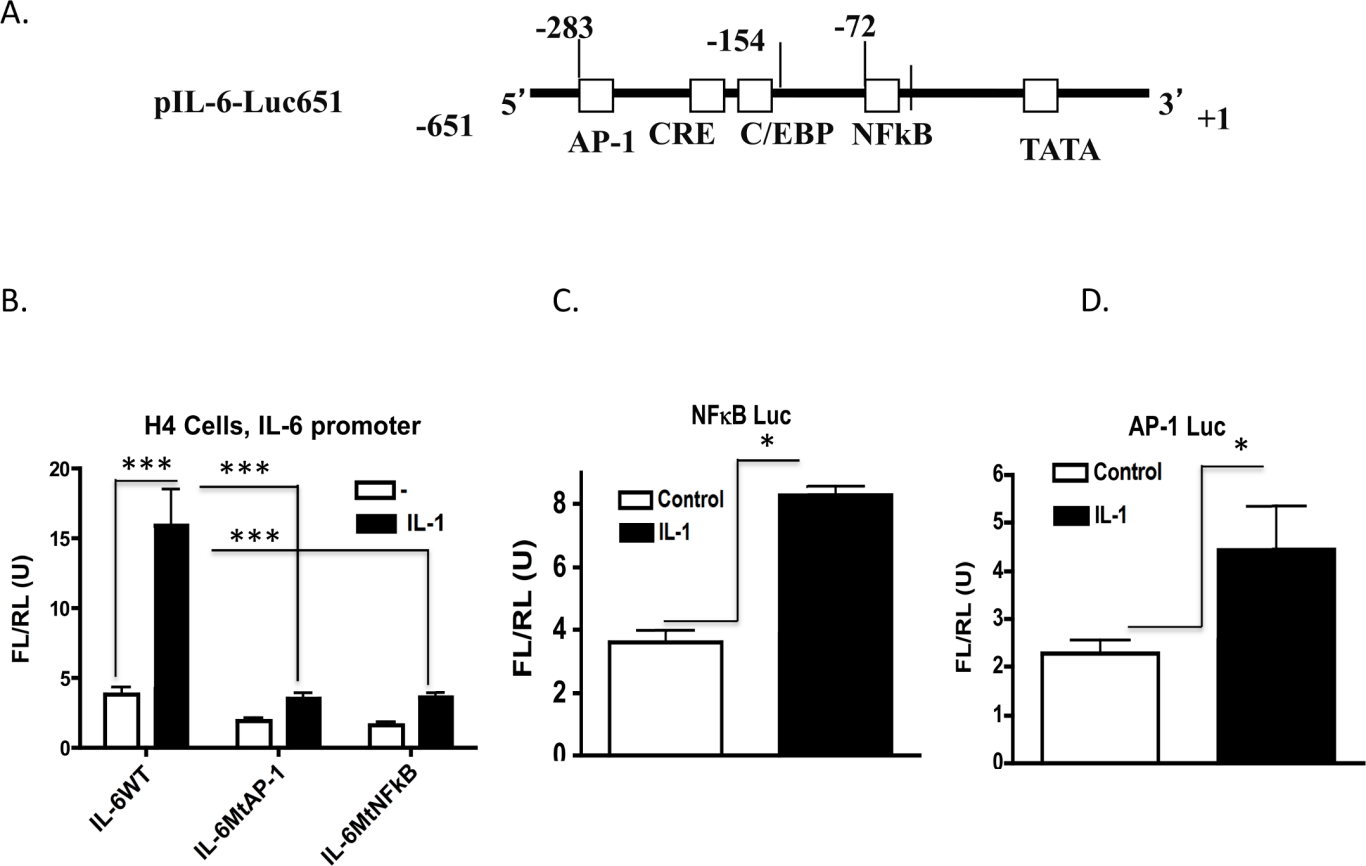
The IL-6 promoter AP-1 and NFĸB sites are equipotent in IL-1β induction of the IL-6 reporter in the immature H4 enterocyte. **(**A) Linear map of the IL-6 promoter (IL-6 651 Luc) reporter showing the AP-1 site (-283), the NFkB site at (-72) and the C/EBP site at -154 from the transcription start site. **(B)** Effect of mutation of the AP-1 and NFκB binding sites on IL-1β induction of the IL-6 promoter. H4 cells were transfected with the IL-6 promoter reporter plasmids, IL-6-651 wild type, (WT) and (IL-6 Mt AP-1) mutated in the AP-1 site and (IL-6Mt NFκB) mutated in the NFκB site (**C)** Effect of IL-1β stimulation on NFκB and (D) AP-1 Luciferase reporter plasmids in immature H4 cells. Reporter plasmids were transfected into H4 cells together with a Renilla luciferase plasmid for estimation of transfection efficiency. After overnight culture, transfectants (n = 3) were either untreated (open bars) or treated with IL-1b (black bars) for 18h and were harvested and assayed for firefly and renilla luciferase. Data is normalized to renilla luciferase and expressed as Means +/-SE (n = 3), control un induced compared to IL-1 induced, ***p<0.001, *p<0.05. A representative experiment of three separate experiments is presented with consistent results.

Our previous work has demonstrated the importance of NFκB signaling in the immature enterocyte to its’ pro-inflammatory phenotype. To investigate its’ relevance to IL-6 production in response to IL-1β we exposed immature H4 cells to the pharmacological inhibitor of the IKK complex, Wedelolactone (S1A Fig). Data demonstrates that the IKK inhibitor lowers IL-6 production in response to IL-1β by 5-fold, which is similar to the effect of the JNK inhibitor SP600125 (Fig 1B). To compare the importance of the IL-6 promoter AP-1 and NFκB sites for their importance to IL-6 gene transcription we performed reporter assays using the IL-6 promoter luciferase construct (IL-6-651 Luc) with individual mutations in the AP-1 and NFĸB binding sites. The IL-6 promoter AP-1 site is located at -283 from the transcription start site and the NFĸB site is located further downstream at -72 (Fig 7A). Using luciferase reporter promoter assays we demonstrate that both the NFĸB and AP-1 sites are equally important in control of IL-6 gene reporter activation in H4 cells (Fig 7B). Transfection of H4 cells with the wild type IL-6 promoter alone demonstrates a 3-fold activation by IL-1b (Fig 7B). Transfection of IL-6 promoter constructs containing mutations in either the AP-1 or the NFĸB sites significantly lowers baseline and reduces the IL-1 response to baseline levels. In Fig 7C/7D we investigate the IL-1β response of the AP-1 and NFĸB sites in isolation. Both reporters give a similar 2-fold induction by IL-1 confirming the importance of both factors in the response to IL-1 in the immature H4 cell.

### Interleukin 1β induction of the IL-6 promoter is mediated by cJun in the immature enterocyte, a repressor role for JunB in the mature enterocyte

**Fig 8:**
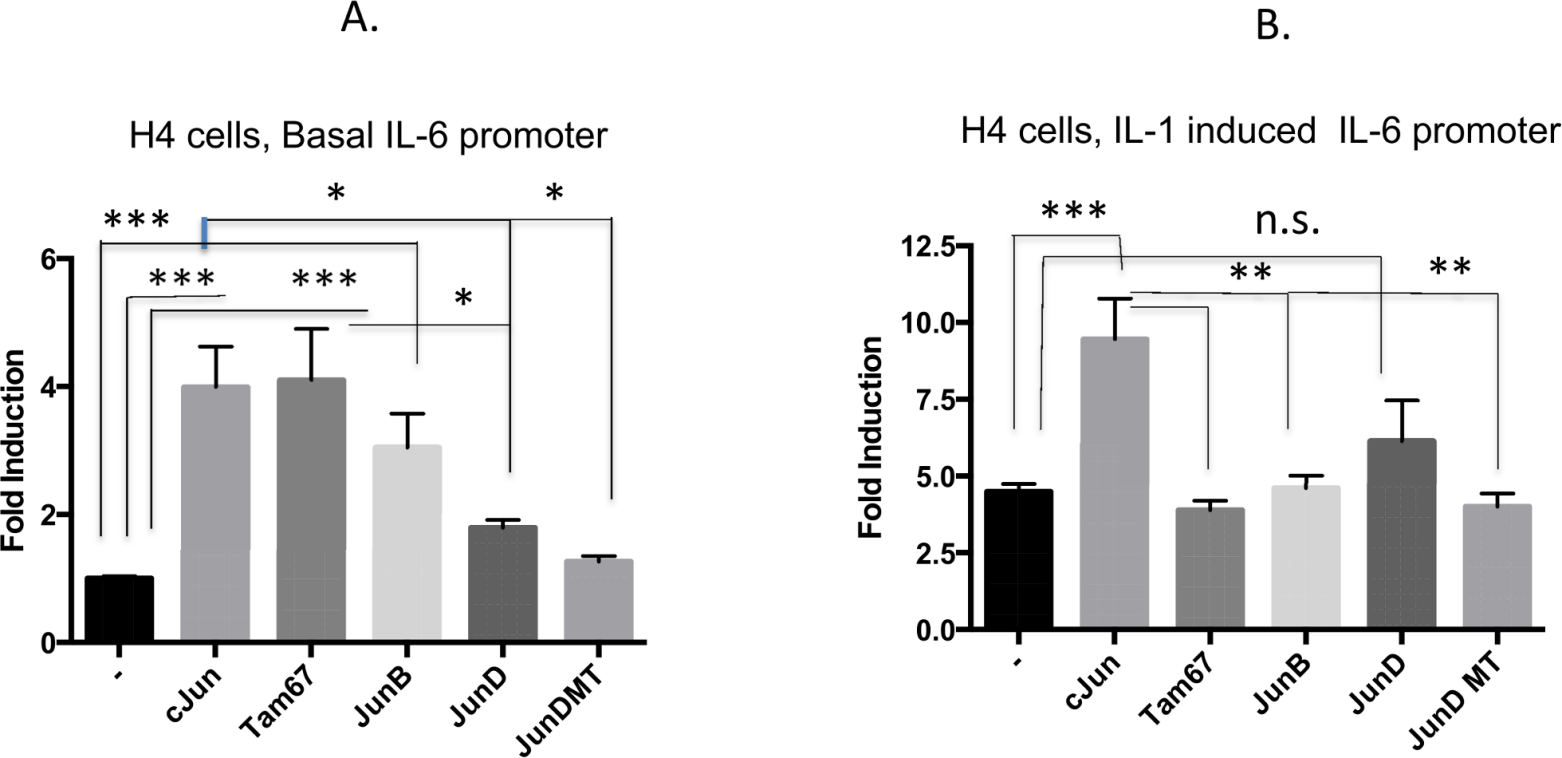
Interleukin 1β induction of IL-6 promoter function is enhanced by cJun in the immature H4 enterocyte. **In A** The IL-6-651 wild type Luciferase plasmid was transfected into H4 cells alone or co-transfected with expression plasmids for cJun, Tam67, JunB JunD, and JunD DN, dominant negative as indicated. A renilla expression plasmid was co-transfected for estimation of transfection efficiency. After overnight culture, serum starved transfectants (n = 3) were either untreated (A) or treated with IL-1b (B) for 18h and were harvested and assayed for firefly and renilla luciferase. Normalized data presented as basal promoter activation (A) or IL-1β induced promoter activation (B) in the presence of empty plasmid control (-) or in the presence of the indicated plasmids. Means +/-SE (n = 3), control un transfected compared to transfected ***p<0.001, **p<0.01, *p<0.05. A representative experiment of three separate experiments is presented with consistent results

**Fig 9:**
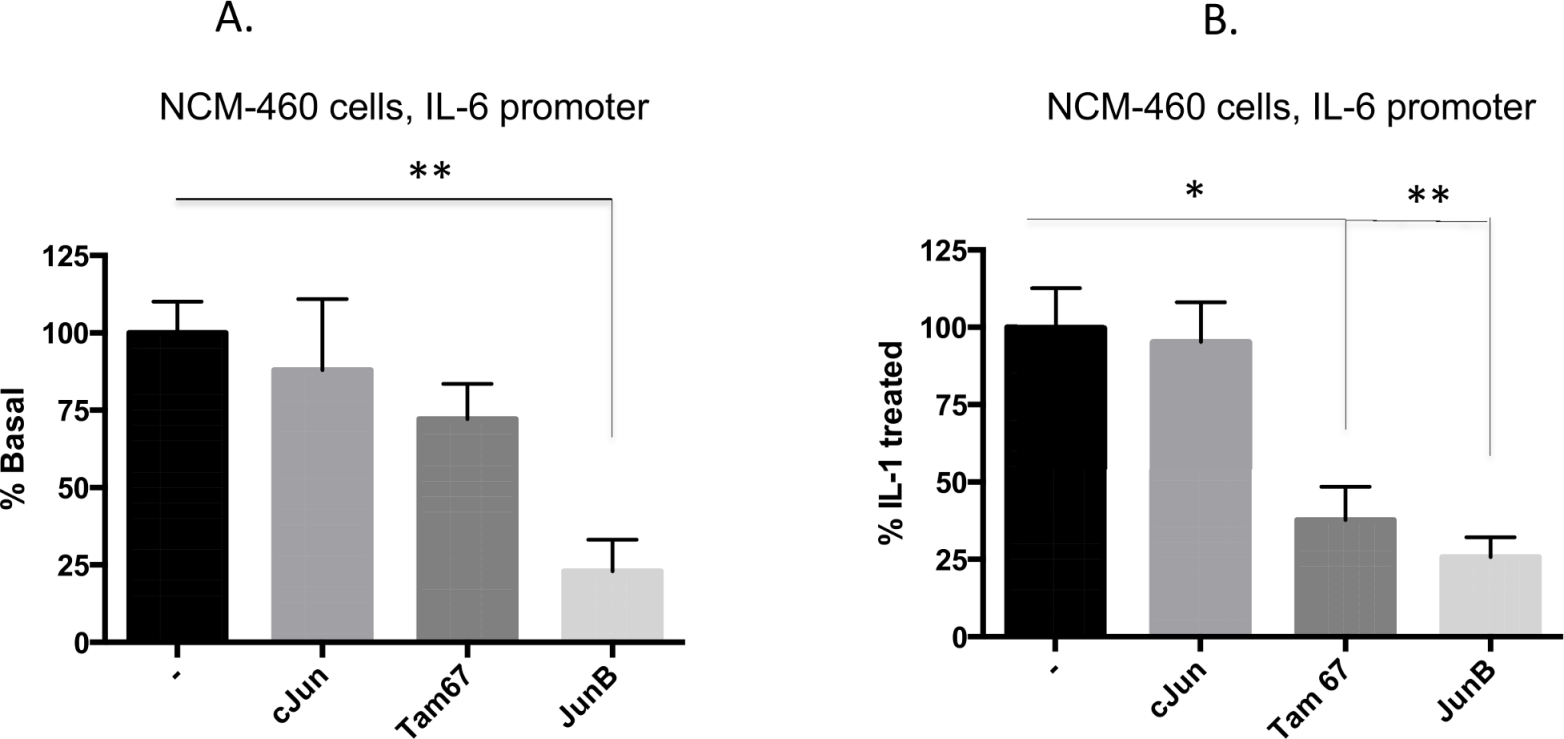
The IL-6 promoter is repressed by JunB in the mature NCM-460 enterocyte. The IL-6-651 luciferase promoter construct was transfected into NCM-460 cells alone or co-transfected with expression plasmids for c Jun, Tam67 or JunB as indicated. A Renilla luciferase plasmid was co-transfected for estimation of transfection efficiency. After overnight culture, transfectants were either untreated (A) or treated with IL-1β (B). Transfectants (n = 3) were harvested and assayed for luciferase. Normalized data, presented as % basal promoter activation (A) or % IL-1β induced promoter activation (B), in the presence of empty plasmid control (-) or in the presence of the indicated plasmids. Data is presented as Means +/-SE (n = 3), un transfected compared to transfected **p<0.01, *p<0.05. A representative experiment of three separate experiments is presented with consistent results.

In order to define the functional role for the Jun family members on IL-6 gene promoter activation in H4 cells we over-expressed these proteins and investigated their effects on both basal (Fig 8A) and IL-1β (Fig 8B) induction of the gene. All of the Jun plasmids expressed similarly (not shown) and significantly induced the IL-6 promoter over baseline levels (including Tam67, the cJun transactivation mutant), however over–expression of JunD was much less effective (2-fold compared to 4 fold) and this was significantly different from that induced by cJun. The almost 5-fold induction of the IL-6 promoter by IL-1β stimulation was further increased to 10 fold by cJun overexpression but was not significantly increased by any other Jun family member including JunD (Fig 8B). In mature NCM-460 cells, the IL-6 promoter assay showed that cJun over-expression neither enhanced basal nor IL-1β induction of the promoter but Tam67 over-expression significantly lowers IL-1β induction suggesting that IL-1β induction of the promoter is also dependent on cJun. Notably however, JunB over-expression significantly lowered baseline and IL-1β induced IL-6 promoter function (Fig 9A and 9B). Taken together, these data support an important activator role for cJun on the IL-6 promoter. A likely cJun antagonistic role for JunB on IL-1β induction of the IL-6 promoter in the colonic NCM-460 enterocyte was observed.

## Discussion

**Fig 10:**
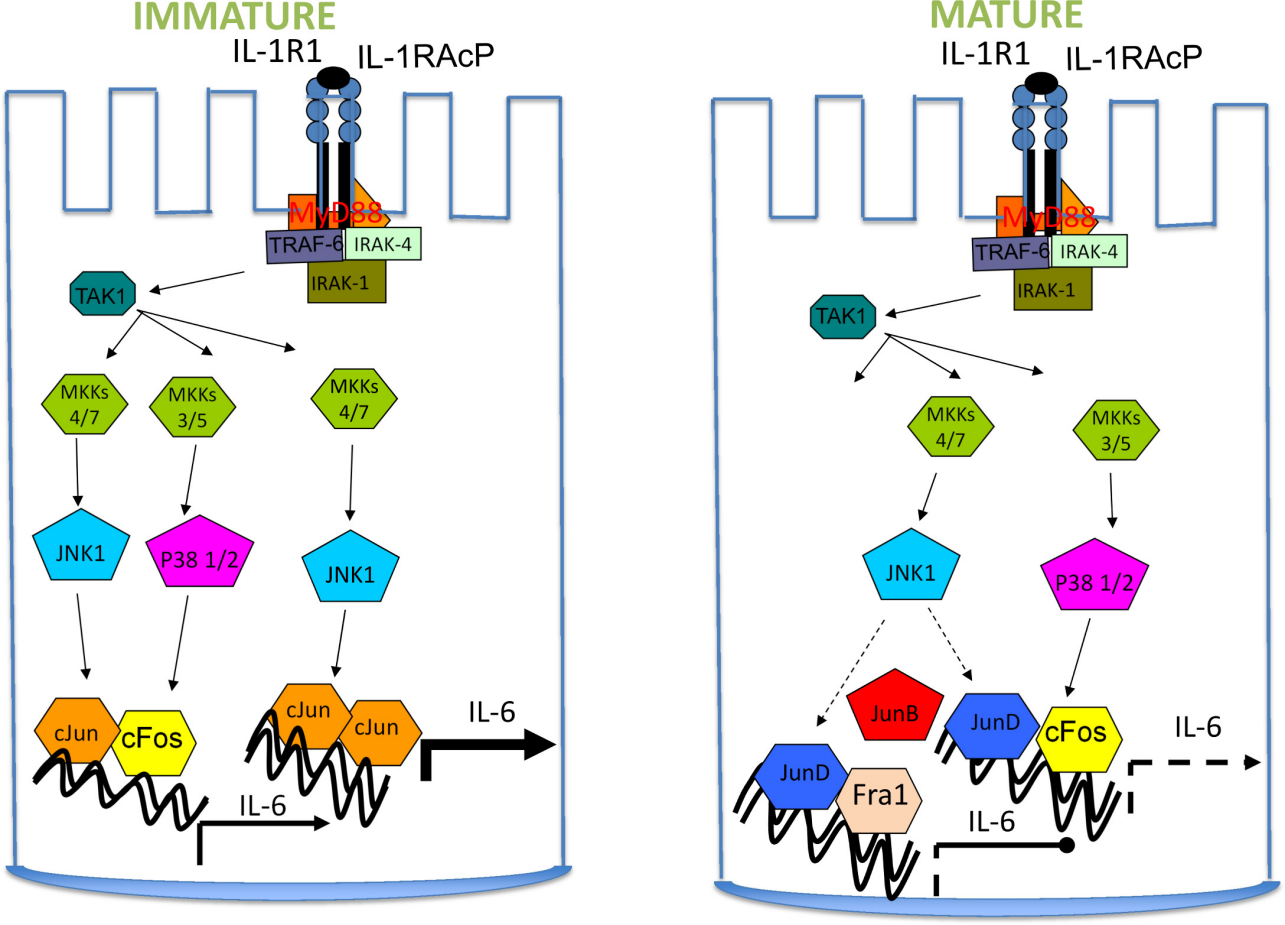
IL-1β induction of IL-6 gene transcription *via* AP-1 in the developing enterocyte. Interleukin 1 (IL-1) binding to the IL-1 receptor (IL-1R1) and co-receptor (IL-1RAc) and formation of the signaling module containing MyD88, IRAK (IL-1 receptor associated kinase) and TRAF6 (TNF receptor associated factor). The Map kinase signaling module is activated, upstream of the Map kinase kinases (MKKs) and Stress Activated Protein kinases (SAPKs) JNK and p38. We have previously shown MyD88 and TRAF-6 mRNA is up regulated in the immature enterocyte [8] Constitutive and IL-1β induced JNK and p38 phosphorylation, together with high expression levels of their target transcription factors, cJun and cFos results in increased IL-6 gene transcription in the immature enterocyte. Expression of JunD later in enterocyte development facilitates formation of the less potent JunD/cJun or JunD/cFos dimers which replace the activating cJun/cJun or cJun/cFos dimers resulting in lower IL-6 gene transcription. A JNK independent pathway involving JunB in the colon represses IL-6 gene transcription.

Despite its’ central role in the regulation of multiple pro-inflammatory cytokine genes there are few studies of AP-1 expression and none to our knowledge on its’ role in inflammatory cytokine gene regulation during human intestinal development. Our data showing elevated canonical AP-1 cJun and cFos in early intestinal development in association with enhanced sensitivity to the pro-inflammatory cytokine interleukin 1β support a role for these immediate early response genes in the development of the intestinal innate immune response [15]. Our data demonstrate the importance of the JNK/cJun pathway in IL-6 gene regulation in the immature enterocyte and decreases in IL-1β was associated with a dramatic decrease in cJun expression, lost responsiveness to JNK, together with an increase in expression of JunD, a Jun family member which is less responsive to JNK. A model emerges whereby an AP-1 dimer switch may occur during intestinal epithelial development from a potent JNK responsive, cJun homodimer or cJun/cFos heterodimer to the less responsive JunD/cFos dimer which may account for the lower enterocyte response to the pro-inflammatory IL-1β and lower IL-6 production in the mature enterocyte (Fig 10).

We have previously shown that IL-1β induction of the pleiotropic cytokine IL-6 is controlled by AP-1 in the mature Caco-2 colonocyte, a colon derived cell that differentiates into enterocytes with small bowel characteristics [14]. However, other DNA-bound transcription factors including NFĸB contribute to the general level of expression and help to establish a multi-protein complex, a so-called ‘enhanceosome’ at the promoter DNA[36]. Our data here demonstrate that both the IL-6 promoter NFĸB and AP-1 sites are equally important in the regulation of the IL-6 gene in immature H4 cells and the pharmacological inhibitor experiments suggest that both factors contribute equally to the pro-inflammatory phenotype of the immature enterocyte. The collaboration between AP-1 and NFĸB on promoters containing both of these binding sites in close proximity is well documented [37]. Important roles for cJun/p65 and Jun B/p65 NFĸB interactions in the control of chemokines and pro-inflammatory cytokines including IL-6 in different cellular contexts have been reported [38,39].

In our *ex vivo* organ culture of human intestinal xenografts, only the very immature (16 week) xenografts had elevated expression of both cFos and cJun and this correlated with their enhanced ability to secrete IL-6 in response to IL-1β stimulation. The villus enterocyte is a rapidly growing cell and it is not surprising we find high expression of cJun, traditionally associated with proliferation [40]. JunD expression appeared later and was observed in cultures derived from xenografts 23 weeks post transplantation when decreases in expression of cJun were observed and lower IL-1β induction of IL-6, showing a decreased pro-inflammatory phenotype. Previous studies in Caco-2 enterocytes, have demonstrated an inverse relationship between cJun and JunD with cJun decreasing and JunD increasing 3 days post confluence [41]. Although our immature H4 cells showed enhanced baseline and IL-1β induced binding of both cJun and JunD to the AP-1 DNA response element we were unable to detect JunD by direct western blotting suggesting that it is unstable in these cells. The H4 cell line used in this laboratory was previously isolated from a 22–23 week old fetus [31,42] and therefore represents an enterocyte derived from a similar aged xenograft (23 weeks) which expresses both cJun and JunD (Fig 6). In terms of IL-6 gene transcription, JunD heterodimers have been previously shown to increase IL-6 transcription while JunD homodimers have a negative effect in different cellular contexts. [43]. The sustained JunD DNA binding in H4 cells preceded by an earlier decrease in cJun binding suggests it may serve to dampen IL-6 gene transcription in the immature H4 cell. Apart from this role, the appearance of JunD in the ileal cultures at 23 weeks is noteworthy as the JunD/JNK pathway has been shown previously to mediate survival signaling. JunD has been shown to inhibit TNF stimulated apoptosis and the JunD/JNK pathway has been shown to co-operate with NFĸB to increase the expression of cIAP-2, an inhibitor of caspases [14,44]. There are several reports of the JunD/cFos dimer associated with cell survival, [45] and It has been proposed that JunD represses cell cycle related genes thereby giving the required time to repair cellular damage prior to proliferation [46]. Therefore with its’ cell survival function in mind, if the timing of induction of JunD is delayed in the developing intestine, critical survival cues may be lost thereby predisposing the immature intestine to inflammation. Indeed our functional data in H4 cells suggests that JunD may have a dampening effect on IL-6 gene transcription. The absence of Jun family members and the increased expression of the non-DNA binding Fos family members in the older post natal xenografts likely contributes to the decreased inflammatory phenotype of the mature enterocytes.

Both JNK and p38 are essential for IL-1β induction of IL-6 in the immature enterocyte and the requirement for JNK is lost with p38 being more essential for IL-6 production as the enterocyte matures. In our H4 enterocytes the transient binding of cJun to the AP-1 response element paralleled the phosphorylation of JNK in response to IL-1β while the binding of JunD did not. The later differences in the binding kinetics of cJun and JunD to the AP-1 response element in response to IL-1β in H4 cells suggest that cJun/JunD may not be a functional heterodimer in these immature cells. The activating dimer in H4 cells is likely the cJun homodimer and it is known that JNK preferentially phosphorylates cJun dimers. cJun homodimers have potential to act as coactivators by recruiting RNA polymerase 2 to chromatin[47] as has been previously reported on the IL-1β promoter itself. In fact cJun is regarded as the most inducible AP-1 transcription factor member. Only cJun is efficiently phosphorylated and stimulated by the JNK pathway because it is the only Jun that contains both an effective docking site and phosphorylation site. While JunB has an efficient JNK docking site it does not have JNK phosphorylation sites and although JunD has JNK phosphorylation sites its’ docking site interacts with JNKs poorly [48].

We observed differential expression of the JNK unresponsive JunB with high expression in colonocytes and in colon derived xenografts, with no expression detected in the ileal xenografts or in the H4 ileal cell line. The high expression of JunB in colon derived, NCM460, Caco-2 and T84 cells (not shown) correlated with low IL-6 secretion from these transformed cell lines supporting its’ role as a transcription repressor of the IL-6 gene. In fact JunB is often considered a transcriptional repressor or a poor activator despite its direct role in the induction of cytokines like IL-2, IL-4, VEGF and cyclin A in various cellular contexts [16,49]. The mechanism is likely associated with its’ antagonism of cJun. It has been reported that the cJun:JunB heterodimer forms 2-fold more readily than the cJun:cJun homodimer and the decreased DNA binding activity together with its preferential formation of hetero-dimers explains why JunB is such an efficient repressor of cJun. The absence of endogenous expression of JunB in the intact ileal xenografts (immature or immature) which could potentially hetero-dimerize with cJun providing anti-inflammatory potential, likely predisposes this area of the intestine to inflammation.

We were unable to detect cFos binding to the AP-1 response element in H4 cells, although a high molecular weight form of cFos was detected by western blotting. This may be a precursor form not capable of dimerization with cJun and therefore not detectable on DNA binding.

Low levels of cFos and cJun binding were detected in NCM-460 mature enterocytes. However elevated binding of another fos family member, FRA1 was detected. It is likely that the activating cJun/cFos dimer is being displaced by repressor dimers containing either JunB or FRA1 as their binding is increased at the later time points following cJun/cFos binding in these lower IL-6 secreting mature cells. It has been shown previously that FRA-1 represses the CEBP/α promoter *via* a promoter proximal AP-1 site [50].

In conclusion, we have discovered an important pattern of AP-1 expression during human intestinal development associated with IL-1β responsiveness and IL-6 gene regulation which suggests that this important transcription factor has a central role in the development of the innate immune response in the intestine. Localization and developmental regulation of Jun family members, in the intestinal epithelium likely plays an important role in the regulation of pro-inflammatory cytokines and provides an avenue for therapeutic intervention for intestinal diseases such as inflammatory bowel disease and necrotizing enterocolitis.

## Supporting Information

S1 Fig Pharmacological inhibition of the IKK complex inhibits IL-6 production in immature H4 cells. Immature H4 cells (A) and mature NCM-460 cells (B) were plated in 24 well plates overnight, incubated in low serum media for 3h and treated with IL-1β (0.5ng/ml) in the absence or presence of the IKK complex inhibitor wedelolactone at the indicated concentrations (triplicate treatments) as indicated. Tissue culture media was harvested after 6 h and assayed for IL-6 by ELISA. Mean +/-S.E (n = 3) from 2 experiments are presented, control un induced compared to IL-1 induced and IL-1 induced compared to IL-1 in the presence of the inhibitors, analyzed by Student T-test.
(PDF)
