# Supplementary figures and images for: Differential Expression of the Activator Protein 1 Transcription Factor Regulates Interleukin-1ß Induction of Interleukin 6 in the Developing Enterocyte
Source: PLoS One. 2016 Jan 22;11(1):e0145184. doi: 10.1371/journal.pone.0145184 (PMC4723075; doi:10.1371/journal.pone.0145184)

Supplementary Figure 1

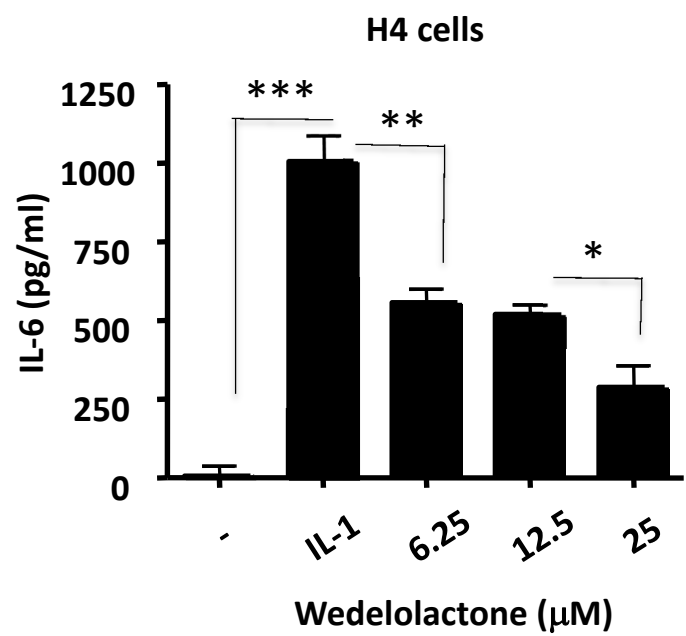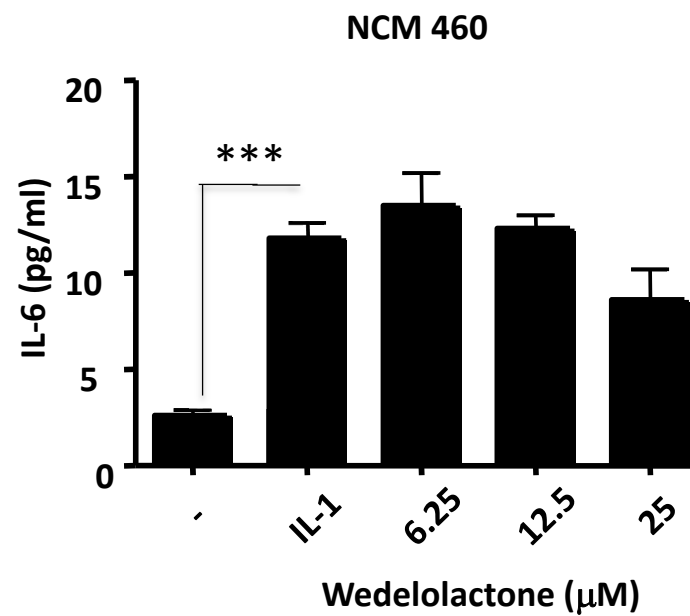

Supplement: S1 Fig — Immature H4 cells (A) and mature NCM-460 cells (B) were plated in 24 well plates overnight, incubated in low serum media for 3h and treated with IL-1β (0.5ng/ml) in the absence or presence of the IKK complex inhibitor wedelolactone at the indicated concentrations (triplicate treatments) as indicated. Tissue culture media was harvested after 6 h and assayed for IL-6 by ELISA. Mean +/-S.E (n = 3) from 2 experiments are presented, control un induced compared to IL-1 induced and IL-1 induced compared to IL-1 in the presence of the inhibitors, analyzed by Student T-test. (PDF) [file pone.0145184.s001.pdf]
